# A Comparison of Isolation Stress and Unpredictable Chronic Mild Stress for the Establishment of Mouse Models of Depressive Disorder

**DOI:** 10.3389/fnbeh.2020.616389

**Published:** 2021-01-07

**Authors:** Jin-Seok Lee, Ji-Yun Kang, Chang-Gue Son

**Affiliations:** Institute of Bioscience & Integrative Medicine, Dunsan Hospital of Daejeon University, Daejeon, South Korea

**Keywords:** depression-psychology, isolation stress, unpredictable chronic mild stress (UCMS), serotonin, microglia, mouse model

## Abstract

This study aimed to help to understand the influence of stress on depression, which reflects the social environments of especially solitary life and the increasing prevalence of depressive disorders. To determine the distinguishable features of two-representative animal models of stress-induced depressive disorder, we compared isolation stress (IS) and unpredictable chronic mild stress (UCMS). After 4-week of stress, both models showed significant depressive- and anxiety-like behaviors in an open field test (OFT; *p* < 0.01 for IS, *p* < 0.01 for UCMS), forced swimming test (FST; *p* < 0.01 for IS, *p* < 0.01 for UCMS), and tail suspension test (TST; *p* < 0.01 for IS, *p* < 0.05 for UCMS) along with alterations in serum corticosterone levels, serotonin activity in the dorsal raphe nuclei (DRN) and microglial activity in the dentate gyrus of the hippocampus (*p* < 0.05 for both parameters). In a comparison of the two stress models, IS strongly induced depressive and anxiety features, as indicated by all parameters: behavior test scores (*p* < 0.05 for OFT, FST, and TST), serum corticosterone levels (*p* < 0.05), immunohistological alterations for serotonin activity (*p* < 0.05) and microglial activity (*p* = 0.072). Our results indicate the suitability of IS for the development of animal models of depressive disorders and may reveal the medical impact of social isolation environment in modern society.

## Introduction

Stressful events are inevitable parts of life in modern society, and they have caused a rapid increase in the number of patients with neuropsychological disorders (Krishnan and Nestler, 2008). Chronic stress would evoke brain structural remodeling (McEwen et al., 2016) and functional loss, including cognition impairments (Lupien et al., 2009) depending on the intensity and/or duration of stress.

To defend against brain damage from stress, glucocorticoids (primary stress response hormones) are secreted in large amounts by hypothalamic-pituitary-adrenocortical (HPA) axis activation. However, their excessive influx into the brain leads to dysfunction of the neurotransmitter system (Lanfumey et al., 2008). Maternally separated rats show low gene expression of the glucocorticoid receptor, which contributes to decreased brain serotonin activity (Meaney et al., 1993; Ladd et al., 2000). Reduced serotonin activity is known as a pathological feature of depressive disorder, and it has been targeted to treat depressive symptoms (Fakhoury, 2016). In a positron emission tomography (PET) imaging study, the observed tendency of a correlation between cortisol and the 5-hydroxytryptamine (5-HT)_1A_ receptor was different under conditions of physical stress (radial artery catheter insertion) vs. psychological stress (the Trier social stress test) in patients with major depressive disorder (Steinberg et al., 2019).

These findings imply that different sensitivities to stress can occur based on the type of stress in the pathogenesis of the depressive disorder. Intriguingly, the severity of chronic stress-derived depressive disorder could be dependent on the specific type of stress, such as physical or emotional stress (Richter-Levin and Xu, 2018). Previous studies have reported that psychological stress, sexual stress, and physical stress lead to different behavioral profiles (Infurna et al., 2016; Hodgdon et al., 2018). To date, various types of stresses are indiscriminately being applied for depressive mouse models. For example, unpredictable chronic mild stress (UCMS) is most commonly used to study depression in rodents (Mineur et al., 2006).

On the other hand, it is well noticed that loneliness from social connectedness leads to stress susceptibility and depressive symptoms, including pessimism, low self-esteem, and anger (Cacioppo et al., 2006). Recently, people who live a solitary life are rapidly rising, and it is closely associated with the increased prevalence of depressive disorder (van den Brink et al., 2018; Achterbergh et al., 2020). To mimic human depression-related disorders, social isolation stress is commonly used (Ieraci et al., 2016). However, no comparative study exists between the isolation stress model and others regarding depression and anxiety disorders.

To figure out the usefulness of the isolation stress model in the aspect of the modern solitary life, we compared two typical stress models, isolation stress vs. UCMS. We investigated the distinguishable features of depressive-like behaviors and depression-related molecules, including serum corticosterone and serotonin in the brain. This study also would help understand the clinical relevance between loneliness and depression.

## Materials and Methods

### Animals and Stress Procedure

Twenty-four specific pathogen-free C57BL/6N male mice (8 weeks old, 22–24 g) were purchased from Dae Han Biolink Co., Limited (Eumseong, South Korea). The mice were housed in plastic cages maintained at 23 ± 1°C with a 12 h light-dark cycle, and they were given free access to food pellets (Cargill Agri Furina, Gyeonggi-do, South Korea) and tap water. After acclimation for 7 days, the mice were randomly divided into three groups (*n* = 8): the non-stress group, the isolation stress (IS) group, and the UCMS groups.

The isolation stress procedure was conducted as previously described (Ieraci et al., 2016). The mice in the IS group were isolated in individual cages (26 × 18 × 13 cm) for 28 days. The UCMS procedure was performed as previously described (Nollet et al., 2013) with slight modifications. Briefly, the mice in the UCMS group were subjected to eight kinds of stress for 28 days: continuous illumination (24 h), wet bedding (24 h), isolation stress (24 h), 45° cage tilting (12 h), food and/or water deprivation (12 h), restraint stress (3 h), 4°C cold stress (1 h), and swimming in cold water (15 min). Mice were divided into two experimental groups (1 - behavioral tests, 2 - immunohistological analysis) to avoid interference from each other. After the final day of stress exposure, the five mice in each group were tested in behavioral tests to assess depressive and anxiety-like behaviors [the open field test (OFT), forced swimming test (FST), and tail suspension test (TST)], and blood samples were collected from the abdominal vein under CO_2_ anesthesia. The blood was left for 30 min at room temperature (RT) to clot, and the serum was separated. To analyze histology, the brains of the remaining three mice in each group were removed under transcardial perfusion with heparin (10 U/ml in PBS) and 4% paraformaldehyde (pH 7.4) and were stored in 4% paraformaldehyde solution.

Animal care procedures and experiments were conducted following the guidelines issued by the Institutional Animal Care and Use Committee of Daejeon University (Daejeon, South Korea; Approval No. DJUARB2020-027) and the Guide for the Care and Use of Laboratory Animals published by the United States National Institutes of Health.

### Anxiety (Open Field Test) and Depressive-Like Behavioral Tests (Forced Swimming Test and Tail Suspension Test)

The open-field test (OFT) was performed as previously described (Gould et al., 2009). The OFT apparatus was a white square plastic box (40 × 40 × 30 cm), and the center area was distinguished by software. Each mouse was placed in the center area of the field, and the time spent in the center zone and locomotor activity were recorded for 5 min in light condition at 100 lux.

The FST was performed as previously described (Can et al., 2012). The FST apparatus consisted of a plastic cylinder (30 × 30 × 50 cm) filled with tap water at 25 ± 1°C to a depth of 23 cm. Each mouse was allowed to swim for 2 min (pre-test), and the immobility, swimming, and climbing time were recorded for 4 min.

The TST was conducted as previously described (Steru et al., 1985). The TST apparatus consisted of a rectangular box (30 × 30 × 50 cm) with a steel bar on the top. After 2 min (pre-test), each mouse was suspended and subsequently, the immobility and activity duration were recorded for 4 min.

All behaviors were recorded using animal behavior tracking software (Smart 3.0, Panlab SL; Barcelona, Spain). The behavioral tests were performed by researchers who were blinded to the experimental conditions.

### Serum Corticosterone Levels

Serum corticosterone levels were measured using an Arbor Corticosterone Enzyme Immunoassay Kit (Ann Arbor, MI, USA) according to the manufacturer’s instructions. The absorbance at 450 nm was measured using a UV spectrophotometer (Molecular Devices, CA, USA).

### Immunohistological Analysis of Iba-1 and 5-HT

For cryoprotection, brains were gradually immersed in 10, 20, and 30% sucrose in 24 h intervals and embedded in OCT compound (Leica Microsystems, Bensheim, Germany) with liquid nitrogen. The brains were cut into frozen coronal sections (30 μm) using a Leica CM3050 cryostat and then stored in a free-floating buffer. For immunohistological analysis, the sections were blocked in 5% normal chicken serum (containing 0.3% Triton X-100) for 1 h. After washing, the sections were incubated with primary antibodies against ionized calcium-binding adaptor molecule 1 (Iba-1, 1:200, 019-19741, Wako) or 5-hydroxytryptamine (5-HT, 1:200, ab66047, Abcam) overnight at 4°C. Subsequently, the sections were incubated with goat anti-rabbit IgG HRP (1:400, ab6722, Abcam) or Alexa Fluor^®^ 488 donkey anti-goat IgG H&L (1:400, ab150129, Abcam) secondary antibodies for 2 h at RT. To analyze the Iba-1-positive cells, the sections were incubated with an avidin-biotin-peroxidase complex (VECTASTAIN ABC kit, Vector Laboratories) for 2 h. The peroxidase activity was visualized using a stable diaminobenzidine solution. To analyze the 5-HT-positive signal, the sections were exposed to DAPI (1:1,000, D9542, Sigma) to stain cell nuclei. Immunoreactivity was observed under an Axio-Phot microscope (Carl Zeiss, Germany). The intensity was quantified using ImageJ 1.46 software (NIH, Bethesda, MD, USA).

### Statistical Analysis

The data are expressed as the mean ± standard deviation (SD). The statistical significance of differences between groups was evaluated by Kruskal–Wallis *H*-test with *post hoc* Mann–Whitney *U*-test using IBM SPSS statistics software, ver. 25.0 (SPSS Inc., Chicago, IL, USA). A value of *p* < 0.05 was considered to indicate statistical significance.

## Results and Discussion

**Figure 1:**
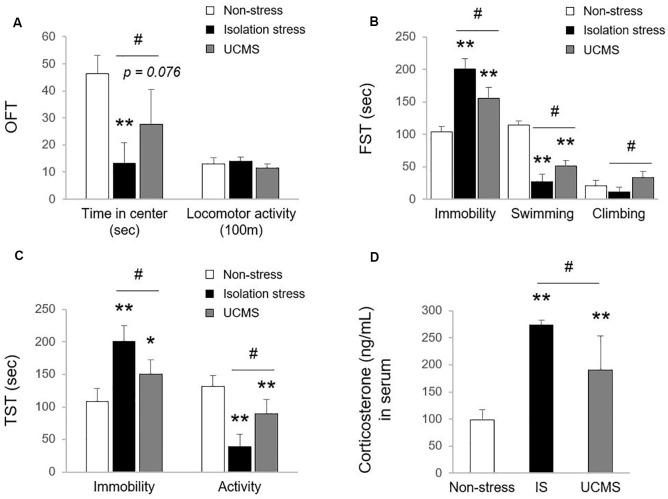
Tests for depressive and anxiety-like behaviors. After exposure to isolation stress (IS) or unpredictable chronic mild stress (UCMS) for 28 days, behavioral tests were consecutively performed. The time spent in the center and locomotor activity in the open field test (OFT; *F*_(2,12)_ = 15.19 for time spent in the center; *p* < 0.01, *F*_(2,12)_ = 2.79 for locomotor activity; **A**), the immobility, swimming, and climbing time in the forced swimming test (FST; *F*_(2,12)_ = 56.99 for immobility; *p* < 0.01, *F*_(2,12)_ = 138.21 for swimming; *p* < 0.01, *F*_(2,12)_ = 8.44 for climbing; **B**) and the immobility and activity duration in the tail suspension test (TST; *F*_(2,12)_ = 21.49 for immobility; *p* < 0.01, *F*_(2,12)_ = 29.61 for activity; *p* < 0.01; **C**) were assessed to analyze depressive and anxiety-like behaviors. The day after the behavioral tests, the levels of corticosterone in serum were evaluated by the enzyme immunoassay method (*F*_(2,12)_ = 26.27; *p* < 0.01 for corticosterone; **D**). The data are expressed as the mean ± SD (*n* = 5). *p < 0.05 and **p < 0.01 compared to the non-stress group, ^#^*p* < 0.05 between the two stress groups.

Uncontrolled and/or repetitive stress is a key contributor to alterations in brain function, which can give rise to the development of the depressive disorder (Richter-Levin and Xu, 2018). Among stress-derived animal models of depressive disorder, social isolation stress (IS; psychological stress) and UCMS (psychophysiological stress) models have been the most commonly used (Mineur et al., 2006; Ieraci et al., 2016). However, there is a lack of comparative study for animal models between IS and UCMS on depression. In our study, 4-week IS and 4-week UCMS both significantly induced depressive- and anxiety-like symptoms, as shown by behavioral tests, including OFT (*p* < 0.01 for IS and *p* = 0.076 for UCMS in time in center, but not in locomotor activity), FST (*p* < 0.01 for both IS and UCMS in immobility and swimming time) and TST (*p* < 0.01 for IS in both immobility and activity duration, *p* < 0.05 and *p* < 0.01 for UCMS in immobility and activity duration, respectively). Compared to UCMS, IS elicited notably worse depression-like behaviors, as evidenced by all three tests, the OFT, FST, and TST (except locomotor activity, *p* < 0.05 for all parameters of behavioral tests; Figures 1A–C). These three behavior tests are well suited to assess depression-related symptoms, including anxiety and despair in rodent models (Gregus et al., 2005; Castagne et al., 2011). One previous study revealed that IS showed a more delayed duration of immobility in defensive burying tests than UCMS-subjected rats, but voluntary ethanol intake was the opposite (Vázquez-León et al., 2017). In general, social withdrawal is known to be more strongly associated with depression and suicidal ideation than work-derived stress (Shin et al., 2017). Many clinical studies have found medical impacts of social isolation on psychiatric disorders; for example, there is a high risk of suicide attempts among people living alone and people who are rejected by their families (Welch, 2001; Yadegarfard et al., 2014; Niu et al., 2020). Early solitude in adolescents is associated with emotional dysfunctions, including low self-esteem, self-harm, and suicidal ideation (Hall-Lande et al., 2007; Wang, 2016; Endo et al., 2017).

**Figure 2:**
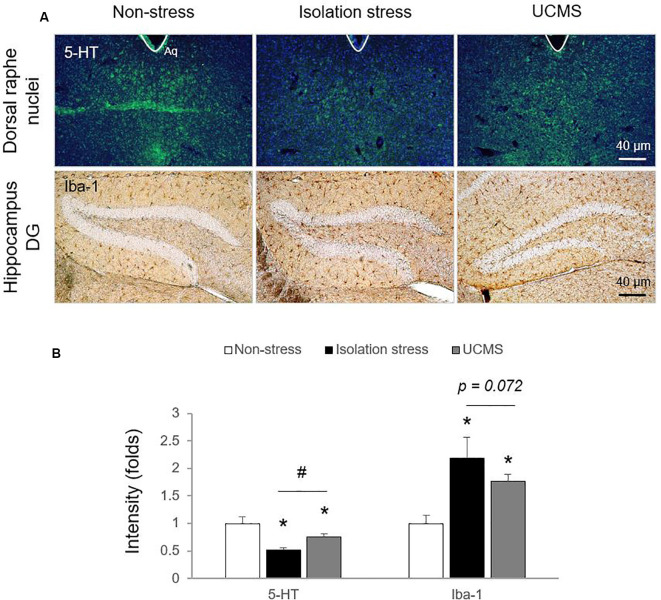
Serotonin levels in the dorsal raphe nuclei (DRN) and microglial activation in the hippocampal dentate gyrus (DG) region. After exposure to isolation stress (IS) or UCMS for 28 days, the brains of mice were prepared for immunohistological analysis. Serotonergic signaling was confirmed by 5-HT immunofluorescence analysis in the DRN (*F*_(2,6)_ = 25.96; *p* < 0.01 for 5-HT), microglial activation was identified by Iba-1 immunohistochemical analysis in the hippocampal DG region (*F*_(2,12)_ = 17.69; *p* < 0.05 for Iba-1; **A**), and the signals were semi-quantified **(B)**. The data are expressed as the mean ± SD (*n* = 3). *p < 0.05 compared to the non-stress group, ^#^*p* < 0.05 between the two stress groups.

In the stress response, glucocorticoids are secreted through HPA axis activation to cope with dangers within minutes (Sapolsky et al., 2000). Hypersecretion of cortisol is well recognized to be involved in the pathology of depressive disorder (Nandam et al., 2019). In patients with depressive disorder, emotional stress triggers dysregulation of HPA axis function and inhibits cortisol metabolism into cortisone, leading to high levels of cortisol (Wust et al., 2000), while physical stress accelerates cortisol metabolism into cortisone (Heijnen et al., 2016). In the present study, compared to 4 weeks of UCMS, 4-weeks of IS resulted in significantly higher serum levels of corticosterone (*p* < 0.05; Figure 1D), which may indicate the superiority of IS for modeling emotional stress-related alterations in corticosterone levels. As expected, we also found molecular alterations in serotonergic and microglial activity, as evidenced by immunohistology against 5-HT and Iba-1 (*p* < 0.05 for both IS and UCMS; Figures 2A,B). It is well-known that chronic social isolation decreases serotonergic activity *via* upregulated ion channel protein (Sargin et al., 2016). The above findings (low serotonergic activity and microglia-derived neuroinflammation) are typical pathological features of depressive disorder (Strickland et al., 2002). Recently, using a PET imaging study, one group revealed that neuroinflammation in emotional-related brain regions in patients with depressive episodes altered the binding of translocator protein (TSPO) to microglia (Setiawan et al., 2015). Furthermore, several studies have suggested that low serotonin activity may be influenced by microglial activation in depressive disorder (Brites and Fernandes, 2015; Glebov et al., 2015). Our data prove that isolation stress causes greater reductions in serotonin in the dorsal raphe nuclei (DRN) and greater activation of microglia in the hippocampal dentate gyrus (DG) region than UCMS (*p* < 0.05 and *p* = 0.072, respectively; Figures 2A,B).

The above results indicate that social isolation more strongly induces depression-related behavior than UCMS. In modern society, individualism is popular, and a large of people are single; these factors are closely associated with a high risk of depressive-like behaviors (Scott et al., 2004; Van Leeuwen et al., 2010). Individuals living alone are known to be vulnerable to depressive disorder (>2-fold more vulnerable than married people; Joutsenniemi et al., 2006). National Psychiatric Morbidity Surveys have shown a proportional relationship between the number of people living alone and the prevalence of mental disorders (Jacob et al., 2019).

Taken together, our results prove the usefulness of isolation stress for developing animal models of depressive disorders. The present study has some limitations, including the relatively small number of sample size in single-gender and only two stress models. Nevertheless, our findings would be also helpful for understanding why interpersonal relationships are important for the prevention of emotional disorders in modern society.

## Data Availability Statement

The original contributions presented in the study are included in the article, further inquiries can be directed to the corresponding author.

## Ethics Statement

The animal study was reviewed and approved by Institutional Animal Care and Use Committee of Daejeon University (DJUARB2020-027).

## Author Contributions

J-SL wrote the main manuscript text, and conducted experiments. J-YK performed the statistical analysis and behavioral test, including tail suspension test, forced swimming test, and open field test. C-GS supervised the manuscript, and directed final version of all contents. All authors reviewed and approved this manuscript. All authors contributed to the article and approved the submitted version.

## Conflict of Interest

The authors declare that the research was conducted in the absence of any commercial or financial relationships that could be construed as a potential conflict of interest.

## References

[B1] Achterbergh L. Pitman A. Birken M. Pearce E. Sno H. Johnson S. (2020). The experience of loneliness among young people with depression: a qualitative meta-synthesis of the literature. BMC Psychiatry 20:415. 10.1186/s12888-020-02818-3 32831064 PMC7444250

[B2] Brites D. Fernandes A. (2015). Neuroinflammation and depression: microglia activation, extracellular microvesicles and microRNA dysregulation. Front. Cell. Neurosci. 9:476. 10.3389/fncel.2015.00476 26733805 PMC4681811

[B3] Cacioppo J. T. Hawkley L. C. Ernst J. M. Burleson M. Berntson G. G. Nouriani B. (2006). Loneliness within a nomological net: an evolutionary perspective. J. Res. Pers. 40, 1054–1085. 10.1016/j.jrp.2005.11.007

[B4] Can A. Dao D. T. Arad M. Terrillion C. E. Piantadosi S. C. Gould T. D. (2012). The mouse forced swim test. J. Vis. Exp. 59:e3638. 10.3791/3638 22314943 PMC3353513

[B5] Castagne V. Moser P. Roux S. Porsolt R. D. (2011). Rodent models of depression: forced swim and tail suspension behavioral despair tests in rats and mice. Curr. Protoc. Neurosci. Chapter 8:Unit 8 10A. 10.1002/0471142301.ns0810as55 21462162

[B6] Endo K. Ando S. Shimodera S. Yamasaki S. Usami S. Okazaki Y.. (2017). Preference for solitude, social isolation, suicidal ideation and self-harm in adolescents. J. Adolesc. Health 61, 187–191. 10.1016/j.jadohealth.2017.02.018 28457686

[B7] Fakhoury M. (2016). Revisiting the serotonin hypothesis: implications for major depressive disorders. Mol. Neurobiol. 53, 2778–2786. 10.1007/s12035-015-9152-z 25823514

[B8] Glebov K. Lochner M. Jabs R. Lau T. Merkel O. Schloss P.. (2015). Serotonin stimulates secretion of exosomes from microglia cells. Glia 63, 626–634. 10.1002/glia.22772 25451814

[B9] Gould T. D. Dao D. T. Kovacsics C. E. (2009). Mood and anxiety related phenotypes in mice. Neuromethods 42, 1–20. 10.1007/978-1-61779-313-4_1

[B10] Gregus A. Wintink A. J. Davis A. C. Kalynchuk L. E. (2005). Effect of repeated corticosterone injections and restraint stress on anxiety and depression-like behavior in male rats. Behav. Brain Res. 156, 105–114. 10.1016/j.bbr.2004.05.013 15474655

[B11] Hall-Lande J. A. Eisenberg M. E. Christenson S. L. Neumark-Sztainer D. (2007). Social isolation, psychological health and protective factors in adolescence. Adolescence 42, 265–286. 17849936

[B12] Heijnen S. Hommel B. Kibele A. Colzato L. S. (2016). Neuromodulation of aerobic exercise-a review. Front. Psychol. 6:1890. 10.3389/fpsyg.2015.01890 26779053 PMC4703784

[B13] Hodgdon H. B. Spinazzola J. Briggs E. C. Liang L. J. Steinberg A. M. Layne C. M. (2018). Maltreatment type, exposure characteristics and mental health outcomes among clinic referred trauma-exposed youth. Child Abuse Negl. 82, 12–22. 10.1016/j.chiabu.2018.05.021 29852362

[B14] Ieraci A. Mallei A. Popoli M. (2016). Social isolation stress induces anxious-depressive-like behavior and alterations of neuroplasticity-related genes in adult male mice. Neural Plast. 2016:6212983. 10.1155/2016/6212983 26881124 PMC4736811

[B15] Infurna M. R. Reichl C. Parzer P. Schimmenti A. Bifulco A. Kaess M. (2016). Associations between depression and specific childhood experiences of abuse and neglect: a meta-analysis. J. Affect. Disord. 190, 47–55. 10.1016/j.jad.2015.09.006 26480211

[B16] Jacob L. Haro J. M. Koyanagi A. (2019). Relationship between living alone and common mental disorders in the 1993, 2000 and 2007 national psychiatric morbidity surveys. PLoS One 14:e0215182. 10.1371/journal.pone.0215182 31042720 PMC6493731

[B17] Joutsenniemi K. Martelin T. Martikainen P. Pirkola S. Koskinen S. (2006). Living arrangements and mental health in finland. J. Epidemiol. Community Health 60, 468–475. 10.1136/jech.2005.040741 16698975 PMC2563935

[B18] Krishnan V. Nestler E. J. (2008). The molecular neurobiology of depression. Nature 455, 894–902. 10.1038/nature07455 18923511 PMC2721780

[B19] Ladd C. O. Huot R. L. Thrivikraman K. V. Nemeroff C. B. Meaney M. J. Plotsky P. M. (2000). Long-term behavioral and neuroendocrine adaptations to adverse early experience. Prog. Brain Res. 122, 81–103. 10.1016/s0079-6123(08)62132-9 10737052

[B20] Lanfumey L. Mongeau R. Cohen-Salmon C. Hamon M. (2008). Corticosteroid-serotonin interactions in the neurobiological mechanisms of stress-related disorders. Neurosci. Biobehav. Rev. 32, 1174–1184. 10.1016/j.neubiorev.2008.04.006 18534678

[B21] Lupien S. J. Mcewen B. S. Gunnar M. R. Heim C. (2009). Effects of stress throughout the lifespan on the brain, behaviour and cognition. Nat. Rev. Neurosci. 10, 434–445. 10.1038/nrn2639 19401723

[B300] McEwen B. S. Nasca D. Gray J. D. (2016). Stress effects on neuronal structure: hippocampus, amygdala, and prefrontal cortex. Neuropsychopharmacology 41, 3–23. 10.1038/npp.2015.171 26076834 PMC4677120

[B22] Meaney M. J. Bhatnagar S. Diorio J. Larocque S. Francis D. O’Donnell D.. (1993). Molecular basis for the development of individual differences in the hypothalamic-pituitary-adrenal stress response. Cell. Mol. Neurobiol. 13, 321–347. 10.1007/BF00711576 8252606 PMC11566811

[B23] Mineur Y. S. Belzung C. Crusio W. E. (2006). Effects of unpredictable chronic mild stress on anxiety and depression-like behavior in mice. Behav. Brain Res. 175, 43–50. 10.1016/j.bbr.2006.07.029 17023061

[B24] Nandam L. S. Brazel M. Zhou M. Jhaveri D. J. (2019). Cortisol and major depressive disorder-translating findings from humans to animal models and back. Front. Psychiatry 10:974. 10.3389/fpsyt.2019.00974 32038323 PMC6987444

[B25] Niu L. Jia C. Ma Z. Wang G. Sun B. Zhang D.. (2020). Loneliness, hopelessness and suicide in later life: a case-control psychological autopsy study in rural china. Epidemiol. Psychiatr. Sci. 29:e119. 10.1017/S2045796020000335 32326999 PMC7214700

[B26] Nollet M. Le Guisquet A. M. Belzung C. (2013). Models of depression: unpredictable chronic mild stress in mice. Curr. Protoc. Pharmacol. Chapter 5:Unit 5:65. 10.1002/0471141755.ph0565s61 23744712

[B27] Richter-Levin G. Xu L. (2018). How could stress lead to major depressive disorder? IBRO Rep. 4, 38–43. 10.1016/j.ibror.2018.04.001 30155523 PMC6111061

[B28] Sapolsky R. M. Romero L. M. Munck A. U. (2000). How do glucocorticoids influence stress responses? Integrating permissive, suppressive, stimulatory and preparative actions. Endocr. Rev. 21, 55–89. 10.1210/edrv.21.1.0389 10696570

[B30] Sargin D. Oliver D. K. Lambe E. K. (2016). Chronic social isolation reduces 5-HT neuronal activity *via* upregulated SK3 calcium-activated potassium channels. eLife 5:e21416. 10.7554/eLife.21416 27874831 PMC5119885

[B31] Scott G. Ciarrochi J. Deane F. P. (2004). Disadvantages of being an individualist in an individualistic culture: idiocentrism, emotional competence, stress and mental health. Aust. Psychol. 39, 143–153. 10.1080/00050060410001701861

[B32] Setiawan E. Wilson A. A. Mizrahi R. Rusjan P. M. Miler L. Rajkowska G.. (2015). Role of translocator protein density, a marker of neuroinflammation, in the brain during major depressive episodes. JAMA Psychiatry 72, 268–275. 10.1001/jamapsychiatry.2014.2427 25629589 PMC4836849

[B33] Shin Y. C. Lee D. Seol J. Lim S. W. (2017). What kind of stress is associated with depression, anxiety and suicidal ideation in korean employees? J. Korean Med. Sci. 32, 843–849. 10.3346/jkms.2017.32.5.843 28378560 PMC5383619

[B34] Steinberg L. J. Rubin-Falcone H. Galfalvy H. C. Kaufman J. Miller J. M. Sublette M. E.. (2019). Cortisol stress response and *in vivo* PET imaging of human brain serotonin 1A receptor binding. Int. J. Neuropsychopharmacol. 22, 329–338. 10.1093/ijnp/pyz009 30927011 PMC6499240

[B35] Steru L. Chermat R. Thierry B. Simon P. (1985). The tail suspension test: a new method for screening antidepressants in mice. Psychopharmacology 85, 367–370. 10.1007/BF00428203 3923523

[B36] Strickland P. L. Deakin J. F. Percival C. Dixon J. Gater R. A. Goldberg D. P. (2002). Bio-social origins of depression in the community. Interactions between social adversity, cortisol and serotonin neurotransmission. Br. J. Psychiatry 180, 168–173. 10.1192/bjp.180.2.168 11823330

[B37] van den Brink R. H. S. Schutter N. Hanssen D. J. Elzinga B. M. Rabeling-Keus I. M. Stek M. L.. (2018). Prognostic significance of social network, social support and loneliness for course of major depressive disorder in adulthood and old age. Epidemiol. Psychiatr. Sci. 27, 266–277. 10.1017/S2045796017000014 28183368 PMC6998855

[B38] Van Leeuwen N. Rodgers R. Regner I. Chabrol H. (2010). The role of acculturation in suicidal ideation among second-generation immigrant adolescents in france. Transcult. Psychiatry 47, 812–832. 10.1177/1363461510382154 21088105

[B39] Vázquez-León P. Martínez-Mota L. Quevedo-Corona L. Miranda-Páez A. (2017). Isolation stress and chronic mild stress induced immobility in the defensive burying behavior and a transient increased ethanol intake in wistar rats. Alcohol 63, 43–51. 10.1016/j.alcohol.2017.03.005 28847381

[B40] Wang J. M. (2016). Preference-for-solitude and depressive symptoms in chinese adolescents. Pers. Individ. Differ. 100, 151–156. 10.1016/j.paid.2015.09.033

[B41] Welch S. S. (2001). A review of the literature on the epidemiology of parasuicide in the general population. Psychiatr. Serv. 52, 368–375. 10.1176/appi.ps.52.3.368 11239107

[B42] Wust S. Federenko I. Hellhammer D. H. Kirschbaum C. (2000). Genetic factors, perceived chronic stress and the free cortisol response to awakening. Psychoneuroendocrinology 25, 707–720. 10.1016/s0306-4530(00)00021-4 10938450

[B43] Yadegarfard M. Meinhold-Bergmann M. Ho R. (2014). Family rejection, social isolation and loneliness as predictors of negative health outcomes (depression, suicidal ideation and sexual risk behavior) among thai male-to-female transgender adolescents. J. LGBT Youth 11, 347–363. 10.1080/19361653.2014.910483

